# Increased Polymerase Activity of Zoonotic H7N9 Allows Partial Escape from MxA

**DOI:** 10.3390/v14112331

**Published:** 2022-10-24

**Authors:** Philipp P. Petric, Jacqueline King, Laura Graf, Anne Pohlmann, Martin Beer, Martin Schwemmle

**Affiliations:** 1 Institute of Virology, Medical Center—University of Freiburg, 79104 Freiburg, Germany; 2 Faculty of Medicine, University of Freiburg, 79110 Freiburg, Germany; 3 Spemann Graduate School of Biology and Medicine, University of Freiburg, 79104 Freiburg, Germany; 4 Institute of Diagnostic Virology, Friedrich-Loeffler-Institute, 17493 Greifswald-Insel Riems, Germany

**Keywords:** influenza, HPAIV, MxA, myxovirus resistance protein A, H7N9, virus evolution, human adaptation

## Abstract

The interferon-induced myxovirus resistance protein A (MxA) is a potent restriction factor that prevents zoonotic infection from influenza A virus (IAV) subtype H7N9. Individuals expressing antivirally inactive MxA variants are highly susceptible to these infections. However, human-adapted IAVs have acquired specific mutations in the viral nucleoprotein (NP) that allow escape from MxA-mediated restriction but that have not been observed in MxA-sensitive, human H7N9 isolates. To date, it is unknown whether H7N9 can adapt to escape MxA-mediated restriction. To study this, we infected Rag2-knockout (Rag2^−/−^) mice with a defect in T and B cell maturation carrying a human MxA transgene (MxA^tg/−^Rag2^−/−^). In these mice, the virus could replicate for several weeks facilitating host adaptation. In MxA^tg/−^Rag2^−/−^, but not in Rag2^−/−^ mice, the well-described mammalian adaptation E627K in the viral polymerase subunit PB2 was acquired, but no variants with MxA escape mutations in NP were detected. Utilizing reverse genetics, we could show that acquisition of PB2 E627K allowed partial evasion from MxA restriction in MxA^tg/tg^ mice. However, pretreatment with type I interferon decreased viral replication in these mice, suggesting that PB2 E627K is not a true MxA escape mutation. Based on these results, we speculate that it might be difficult for H7N9 to acquire MxA escape mutations in the viral NP. This is consistent with previous findings showing that MxA escape mutations cause severe attenuation of IAVs of avian origin.

## 1. Introduction

Influenza A viruses (IAVs) predominantly circulate in wild aquatic birds but occasionally spread to other species [1], including mammals such as pigs and humans. The zoonotic nature of IAV is a threat to the human population, since such infection events are often lethal and, in rare cases, can result in the emergence of a new pandemic virus [2]. However, zoonotic IAVs must adapt to various human host factors to replicate efficiently in the new host, including the evasion from restriction factors [3,4,5]. One potent restriction factor of IAVs is the interferon-stimulated, human myxovirus resistance protein A (MxA) [6,7]. We have previously shown that the origin of the viral NP determines the sensitivity to MxA-mediated antiviral restriction [8]. While avian IAVs are very sensitive to MxA, human-adapted IAVs are largely resistant. This resistance could be linked to specific amino acid substitutions in NP which are absent in avian IAV strains [9]. For the 1918 Spanish Flu three amino acids, namely 100I/V, 283P, and 313Y are sufficient to mediate MxA resistance [9]. A similar but independently evolved set of amino acids (53D, 100I/V, 313V) was observed for the pandemic H1N1 virus of 2009 [9]. These sets of amino acids are only found in human IAV strains and are absent in all avian IAVs, most likely due to the absence of an antivirally active Mx protein in avian species. Recently, selection of a new set of Mx1 resistance enhancing amino acids (48Q, 98K, and 99K) was observed in the Eurasian avian-like swine influenza A lineage, circulating exclusively in pigs. These mutations represent an adaptation to porcine Mx1 (poMx1), which has similar, albeit weaker, antiviral activity compared to human MxA [10]. Interestingly, they also confer partial resistance to human MxA [10].

Artificial introduction of the Mx-escape mutations of the 1918 pandemic virus into avian IAVs of the H5N1 or H7N7 subtype caused impaired viral fitness that could only be compensated by an additional adaptive mutation in NP (16D) which itself does not contribute to MxA resistance [11]. However, combination of the MxA-resistance enhancing amino acids identified in the 1918 pandemic virus (100V, 283P, 313Y) with 16D significantly increased the MxA-resistance of avian IAVs in vitro and in vivo without fitness loss [11,12].

In addition to these amino acids enhancing MxA-resistance, increased viral replication capacity has been proposed as a second mechanism how IAVs can overcome Mx-mediated restriction [13]. High replication capacities allowed IAVs to partially evade restriction by the murine Mx1 protein. However, with all known human, seasonal and pandemic IAV strains encoding MxA-resistance enhancing amino acids in NP, this second evasion strategy does not seem to be sufficient to escape from MxA.

Nevertheless, avian IAVs, like the H5N1 and H7N9 subtypes, have repeatedly crossed the species barrier and infected humans [2]. However, sequencing of human virus isolates did not reveal previously described MxA escape mutations in the viral genome [14]. This could be partially explained by the observation that roughly 7% of the patients infected with H7N9 are heterozygous carriers of rare variations in the human *MX1* gene that lead to the expression of inactive MxA variants with a dominant negative effect on wildtype MxA [15]. Whether antivirally active MxA was expressed in patients lacking these rare mutations in the *MX1* gene is not known. Thus, the absence of MxA escape mutations in H7N9 viruses isolated from humans might be due to the absence of an antivirally active MxA or due to the inability of H7N9 to acquire such mutations without the loss of viral fitness.

To test whether H7N9 is cabable to acquire MxA escape mutations, we infected MxA-expressing Rag2^−/−^ mice (MxA^tg/−^Rag2^−/−^). Rag2^−/−^ mice are a suitable model since they have been shown to allow prolonged IAV infection [16]. In these mice, viral clearance is prevented due to the lack of mature B and T cells [17]. Therefore, MxA^tg/−^Rag2^−/−^ mice allow the study of viral adaptation to host factors because prolonged infection enables the continuous acquisition of mutations in the viral genome.

## 2. Materials and Methods

### 2.1. Cells

Canine MDCK-II, MDCK-SIAT1, MDCK-SIAT1-MxA, and MDCK-SIAT1-MxA-T103A cells [18], as well as, human HEK293T cells were maintained with DMEM supplemented with 10% fetal calf serum, 2 mM L-glutamine, and 1% penicillin-streptomycin.

MDCK-SIAT1 cells express the human 2,6-sialtransferase (SIAT1) and allow efficient infection with IAV with human receptor specificity. These cells were transduced with MxA or MxA-T103A transgenes under the control of a CMV promoter as described in [18] and have previously been used to study MxA resistance phenotypes of IAV [10]. The cells were kept at 37 °C and 5% CO_2_ in a tissue culture incubator.

### 2.2. Plasmid Construction

Full-length H7N9 (A/Guangdong/17SF003/2016) segment sequences were synthesized by Biocat (Heidelberg, Germany) and cloned into the pHW2000 vector using BsmBI or into the pCAGGS vector using NotI and XhoI restriction enzymes. The PB2 E627K mutation was introduced into PB2 segments in both vectors via QuikChange PCR or two-step assembly PCR.

### 2.3. Generation of Recombinant Influenza A Viruses

Recombinant wildtype (wt) and mutated A/Guangdong/17SF003/2016 (H7N9) were generated using the well-established eight-plasmid reverse genetics system [19]. Plaque purification as well as generation of viral stocks were performed on MDCK-II cells. The presence or absence of the PB2 E627K mutation was confirmed by Sanger sequencing of PB2 cDNA prepared via One-Step RT-PCR kit (Qiagen, Hilden, Germany) from isolated viral RNA.

### 2.4. Polymerase Reconstitution Assays

Polymerase activity of the authentic A/Guangdong/17SF003/2016 (H7N9) polymerase with and without the PB2 E627K substitution was analyzed via polymerase reconstitution assays. HEK293T cells were seeded into 12-wells and transfected with pCAGGS plasmids encoding the viral polymerase subunits (10 ng each), viral NP (100 ng), and the artificial viral minigenome construct pPolI-FFLuc-RT (100 ng) encoding firefly luciferase in negative sense orientation as a reporter. Transfection efficiency was controlled by cotransfection of 30 ng pRL-SV40 (Promega, Madison, WI, USA) for constitutive expression of *Renilla* luciferase. Additionally, 200 ng of pCAGGS plasmids expressing either MxA or the inactive MxA-T103A mutant [20] or the empty vector as control were transfected. The cells were lysed 24 h post-transfection to measure firefly and *Renilla* luciferase activities using a dual-reporter assay kit (Promega, Madison, WI, USA) according to the manufacturer’s protocol.

### 2.5. Virus Infection in Cell Culture

MDCK-SIAT1, MDCK-SIAT1-MxA, and MDCK-SIAT1-MxA-T103A cells were seeded in 6-well plates and infected with the indicated viruses at a multiplicity of infection (MOI) of 0.001 in 2 mL infection medium (DMEM supplemented with 0.2% bovine serum albumin [BSA] and 1% penicillin-streptomycin). Viral titers at the indicated timepoints were determined via plaque assay.

### 2.6. Animal Experiments

All animal experiments were performed in compliance with the German animal protection law (TierSchG). The mice were housed and handled in accordance with good animal practices as defined by FELASA and the national animal welfare body GV-SOLAS. The animal welfare committees of the University of Freiburg, as well as the local authorities (Regierungspräsidium Freiburg, Freiburg, Germany), approved all animal experiments (Az. 35-9185.81/G-19/05). C57BL/6N mice were obtained from Janvier (Le Genest-Saint-Isle, France). Human MxA-transgenic C57BL/6N mice [12] with and without functional Rag2 were bred locally. Mouse genotypes were confirmed via the KAPA HotStart mouse genotyping kit (KAPA biosystems, Boston, MA, USA) and gene-specific primers. Mice were intraperitoneally (i.p.) anesthesized with a mixture of ketamine (100 µg per g bodyweight) and xylazine (5 µg per g bodyweight) and intranasally (i.n.) infected with the indicated doses of the respective viruses in 40 µL PBS. The organs were harvested at the indicated timepoints and homogenized in 800 µL PBS. Virus titers were determined via plaque assay.

### 2.7. Next-Generation Sequencing

Full-genome sequencing of selected samples from the animal trials was conducted utilizing universal IAV amplification (SuperScript™ III One-Step RT-PCR System with Platinum Taq, Invitrogen, Waltham, MA, USA) followed by sequencing on an Illumina iSeq platform. After amplification and a succeeding clean-up of the PCR products with magnetic AMPure XP Beads (Beckman-Coulter, Brea, CA, USA), library preparation took place with the QIAseq FX DNA Library UDI Kit (Qiagen, Hilden, Germany) according to the manufacturer’s instructions. Quantification of the prepared libraries was conducted with the QIAseq Library Quant Assay Kit (Qiagen, Hilden, Germany) prior to final pooling. An iterative map-to-reference approach of the sequencing data allowed full-genome production of the samples and further frequency analysis of adaptive mutations in Geneious Prime V.2021.0.1 (Biomatters Ltd., Auckland, New Zealand).

## 3. Results

### 3.1. H7N9 Is Highly Sensitive to MxA Restriction

**Figure 1:**
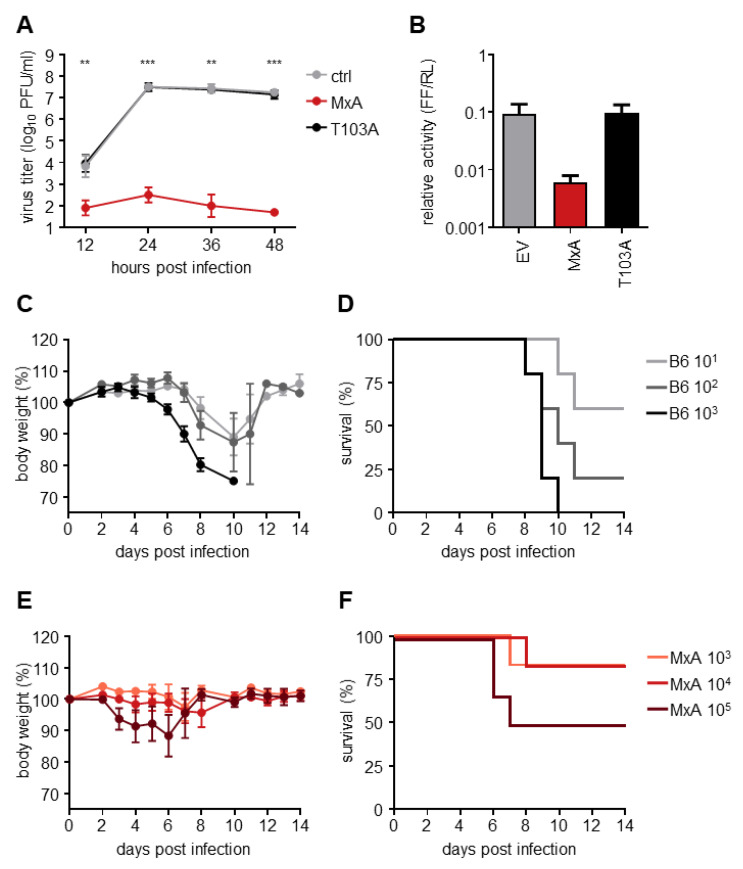
HPAIV H7N9 shows high sensitivity to the antiviral activity of human MxA. (**A**) MDCK-SIAT1 cells (ctrl) and MDCK-SIAT1 cells either expressing antivirally active MxA or inactive MxA-T103A were infected with H7N9 at an MOI of 0.001. Viral titers were determined at the indicated time points via plaque assay. Significance levels indicate differences between MDCK-SIAT1-MxA and -MxA-T103A cells. (**B**) HEK293T cells were co-transfected with expression plasmids coding for the viral polymerase subunits H7N9-PB2, -PB1 and -PA (10 ng each), H7N9-NP (100 ng), an artificial minigenome encoding firefly luciferase as a reporter under the control of the viral promoter (100 ng), and a plasmid encoding Renilla luciferase (30 ng). Constitutive co-expression of Renilla luciferase allows for normalization of transfection efficiency. In addition, expression plasmids encoding either MxA or MxA-T103A (200 ng) or the empty vector (EV) as a control were co-transfected. Luciferase activities were measured 24 h post-transfection. (**C**,**D**) C57BL/6 (B6) and (**E**,**F**) MxA^tg/tg^ (MxA) mice (n = 5 each) were intranasally infected with the indicated doses of H7N9 in 40 µL PBS. Bodyweight changes (**C**,**E**) and survival rates (**D**,**F**) were monitored for 14 days. Mice were sacrificed once their weight fell below 75% of the initial body weight. Student‘s *t* test was performed to determine statistical differences. **, *p* < 0.01; ***, *p* < 0.001.

To analyze the MxA resistance phenotype of H7N9, infection experiments with highly pathogenic avian IAV (HPAIV) A/Guangdong/17SF003/2016 isolated from an H7N9 patient were performed using MxA-expressing cell lines and MxA transgenic mice. Viral growth kinetics in vitro were performed on MDCK-SIAT1 cells expressing either antivirally active MxA or the antivirally inactive MxA-T103A mutant (Figure 1A). At 24 h post-infection (hpi) viral growth peaked with titers > 10^7^ PFU/mL on both control and MxA-T103A-expressing cells. On wt MxA-expressing cells, however, viral growth was significantly reduced compared to MxA-T103A-expressing cells, with titers < 10^3^ PFU/mL at all indicated timepoints. Moreover, in the polymerase reconstitution assay, the relative activity of the authentic H7N9 polymerase was reduced ~15-fold in the presence of MxA wt compared to MxA-T103A or the empty vector control (Figure 1B). To confirm the high MxA-sensitivity of H7N9 in vivo, the LD_50_ of this isolate was measured in human MxA-transgenic mice (MxA^tg/tg^), as well as in C57BL/6 (B6) mice, which naturally lack antivirally active Mx proteins. Mice were infected with different doses of inoculum, depending on their genotype. MxA^tg/tg^ mice were infected with 10^3^, 10^4^ or 10^5^ PFU, whereas B6 mice were infected with 10^1^, 10^2^ or 10^3^ PFU. A dose-dependent effect on weight loss and survival was observed for both genotypes (Figure 1C–F). The LD_50_ was calculated to be approximately 5 × 10^4^ PFU in MxA^tg/tg^ and 2 × 10^1^ PFU in B6 mice using the Reed-Münch method [21]. Taken together, H7N9 A/Guangdong/17SF003/2016 was highly restricted in mice expressing human MxA, confirming the in vitro data described above and previous findings from infection experiments in MxA^tg/tg^ mice with LPAIV H7N9 [12].

### 3.2. Rag2^−/−^ Mice Sustain Prolonged Infection with H7N9

**Figure 2:**
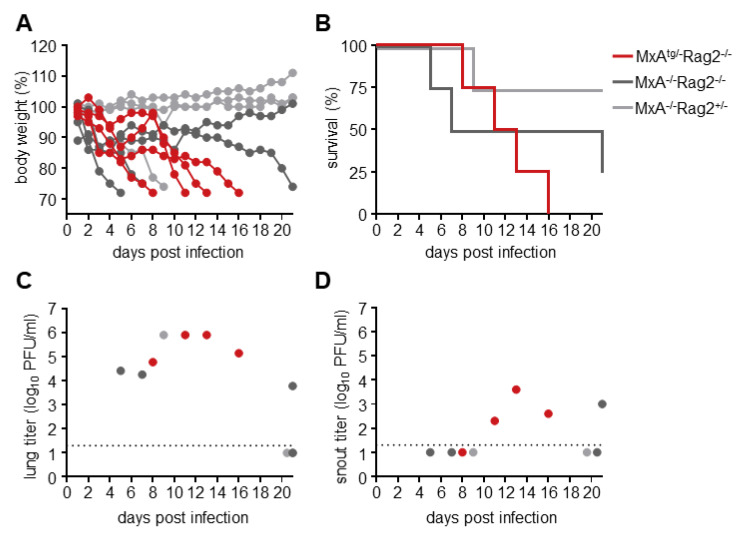
Rag2^−/−^ mice support prolonged IAV infection. (**A**,**B**) MxA^tg/−^Rag2^−/−^ mice and MxA^−/−^ mice with and without functional Rag2 (n = 4 each) were infected intranasally with 40 µL of 0.2 × LD_50_ depending on their MxA genotype (10^4^ PFU for MxA^tg/−^ and 4 PFU for MxA^−/−^). Body weight changes (**A**) and survival rates (**B**) were monitored for 21 days. Mice were sacrificed once their weight fell below 75% of the initial body weight. At the day of death (or on day 21 for the surviving mice) lungs (**C**) and snouts (**D**) were harvested to determine viral titers and to isolate virus for further analysis.

To allow the acquisition of possible MxA-escape mutations due to prolonged replication, MxA^tg/−^Rag2^−/−^ mice (n = 4) were infected with 0.2 × LD_50_ equaling 10^4^ PFU of the H7N9 virus (Figure 2A,B). In parallel, MxA-negative mice, with a heterozygous or knock-out genotype for Rag2 (MxA^−/−^Rag2^+/−^ [n = 4] and MxA^−/−^Rag2^−/−^ [n = 4]), were infected with 0.2 × LD_50_ equaling 4 × 10^0^ PFU. One MxA^tg/−^Rag2^−/−^ and one MxA^−/−^Rag2^−/−^ mouse reached the critical weight loss of >25% of initial body weight at 16 and 21 days post-infection (dpi), respectively, indicating a highly prolonged infection. In contrast to the MxA^tg/tg^ mice, which efficiently cleared the viral infection through their adaptive immune response, the Rag2^−/−^ mice (regardless of their MxA genotype) were expected to eventually succumb to the infection due to the lack of an adaptive immune response. Indeed, all of the MxA^tg/−^ mice with Rag2^−/−^ backgrounds succumbed to the infection. The MxA^−/−^Rag2^+/−^ mice and only one of the MxA^−/−^Rag2^−/−^ animals infected with the low dose of H7N9 recovered during the experiment. The lung and snout of the infected animals were harvested on the day of death (or on day 21 for the surviving mice) to determine the viral titers in these organs (Figure 2C,D). Except for the recovered mice, viral titers of up to 10^6^ PFU/mL were measured in the lungs of all animals. In some of the infected animals, virus was also detectable in the snouts although titers were much lower (Figure 2D).

### 3.3. Viruses Isolated from MxA^tg/−^Rag2^−/−^ Mice Show Enhanced Growth in MxA^tg/tg^ Animals

**Figure 3:**
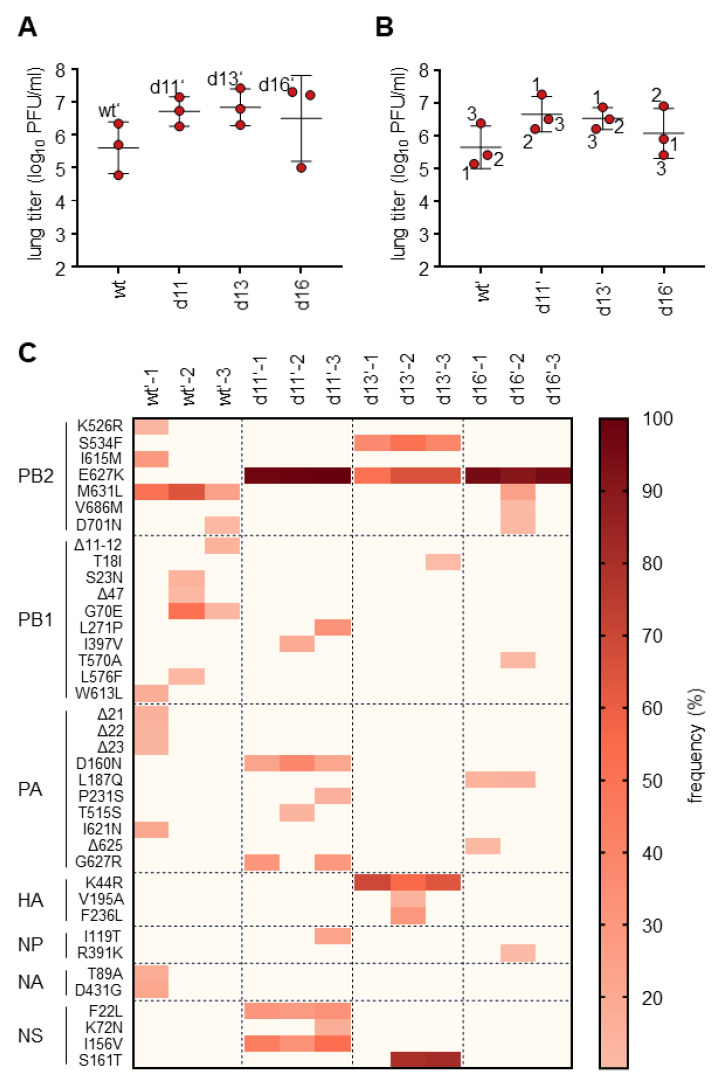
Virus isolates from MxA^tg/−^Rag2^−/−^ mice show enhanced replication in MxA^tg/tg^ mice. (**A**) MxA^tg/tg^ mice were infected intranasally with 10^4^ PFU of the viral lung isolates of day 11, day 13, and day 16 in Figure 2 or wt H7N9 as a control. The lungs of the infected mice were harvested 3 dpi to determine viral titers. (**B**) MxA^tg/tg^ mice were infected intranasally with 10^4^ PFU of the viral isolates with the highest titers in each group from the experiment shown in (**A**) (indicated with ′). The lungs of the infected mice were harvested 3 dpi to determine viral titers via plaque assay. (**C**) The twelve isolates from (**B**) were subjected to full-genome sequencing for frequency analysis of adaptive mutations. All mutations that were found with a frequency of at least 10% are listed. HA mutations are displayed according to H7N7 numbering [23].

To analyze whether the viruses isolated from MxA^tg/−^Rag2^−/−^ mice show increased MxA resistance in vivo, the viruses isolated at days 11, 13, and 16 post-infection (d11, d13, d16; Figure 2C) were used to infect MxA^tg/tg^ mice with 10^4^ PFU (n = 3 for each virus; Figure 3A). Lung titers were analyzed 3 dpi and compared to the parental H7N9 virus stock (wt). Although the observed lung titers were quite heterogeneous, the virus isolates of MxA^tg/−^Rag2^−/−^ mice were able to reach titers of up to 10^7^ PFU/mL, whereas lower titers were observed with the original H7N9 virus stock. Due to low animal numbers (n = 3) and the high variance in titers between animals, no statistical significance could be observed. A second round of infection in MxA^tg/tg^ mice was performed with lung homogenates derived from the animals with the highest lung titers in each group (Figure 3A). These isolates were termed wt′, d11′, d13′, and d16′. As in the first round of infection, heterogeneous lung titers were observed for wt′, d11′, d13′ as well as d16′, suggesting the presence of a mixture of viral variants (Figure 3B).

To analyze which mutations responsible for the enhanced growth in the MxA^tg/tg^ mice arose in these isolates, all 12 isolates depicted in Figure 3B were subjected to full-genome sequencing and compared to the wt H7N9 stock. Mutations were found in all viral segments except for segment 7 encoding the M1 and M2 proteins (Figure 3C). Most of the identified mutations were present either in only one virus isolate (e.g., in d13′-3: PB1-T18I) or at low frequency in all three isolates derived from infections with the same original lung homogenate (e.g., d11′-1-3: PA-D160N). However, one mutation, PB2-E627K was present at higher frequencies in all isolates passaged in MxA^tg/−^Rag2^−/−^ mice, but not in the isolates derived from infection of MxA^tg/tg^ mice with the parental H7N9 virus stock (wt′) (Figure 3C). This amino acid exchange has previously been described as an adaptive mutation in mammals [22]. Interestingly, another previously described mammalian adaption mutation in PB2, M631L, was also detected in all wt′ isolates at frequencies ranging from 24.1% to 64%. Other mutations in PB2 (S534F), PB1 (G70E), PA (D160N, L187Q, and G627R), HA (K44R, H7N7 numbering system [23]), and NS1 (F22L, I156V, and S161T) were detected at high frequencies in several isolates.

### 3.4. The Mutation PB2 E627K Leads to a Growth Advantage in MxA-Expressing In Vitro and In Vivo Models

**Figure 4:**
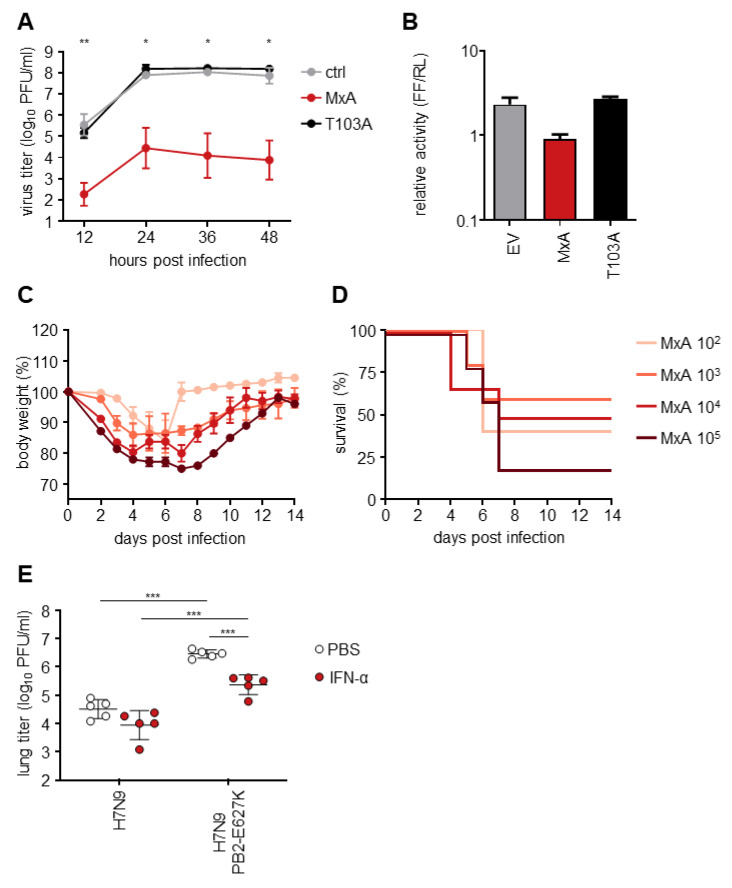
The PB2 E627K mutation enhances viral replication of HPAIV H7N9. (**A**) MDCK-SIAT1 cells (ctrl) and MDCK-SIAT1 cells either expressing antivirally active MxA or inactive MxA-T103A were infected with H7N9 at an MOI of 0.001. Viral titers were determined at the indicated time points via plaque assay. Significance levels indicate differences between MDCK-SIAT1-MxA and -MxA-T103A cells. (**B**) Viral polymerase reconstitution assay were performed as described in the legend to Figure 1B using a plasmid encoding PB2-E627K instead of wildtype PB2. (**C**,**D**) MxA^tg/tg^ mice (MxA) were infected intranasally with the indicated doses of H7N9-PB2-E627K in 40 µL PBS. Body weight changes (**C**) and survival rates (**D**) were monitored for 14 days. Mice were sacrificed once their weight fell below 75% of the initial body weight. (**E**) MxA^tg/tg^ mice were pretreated with IFN-α (105 units) or PBS 24 h prior to infection with 10^4^ PFU of the indicated viruses. The lungs of the infected mice were harvested 3 dpi to determine viral titers. Student‘s *t* test was performed to determine statistical differences. *, *p* < 0.05; **, *p* < 0.01; ***, *p* < 0.001.

To analyze the influence of PB2 E627K on the escape from MxA-dependent restriction, we generated a recombinant A/Guangdong/17SF003/2016 virus expressing PB2-627K (H7N9-PB2-E627K) by reverse genetics. Introduction of the amino acid exchange E627K in PB2 resulted in an approximately 10-fold increase of viral growth in both MDCK-SIAT1 control and MxA-T103A-expressing cells (Figure 4A) compared to the wt virus (Figure 1A). In MxA-expressing cells, H7N9-PB2-E627K replicated to peak titers of 10^5^ PFU/mL (Figure 4A) in contrast to the drastically reduced viral growth of wt H7N9 (Figure 1A). Similarly, the viral polymerase activity in the presence of the amino acid substitution PB2-E627K was reduced only 3-fold when MxA was co-expressed (Figure 4B), compared to a 15-fold reduction of the wt polymerase activity (Figure 1B). Consistently, increased weight loss and decreased survival was observed for H7N9-PB2-E627K-infected MxA^tg/tg^ mice (Figure 4C,D), compared to mice infected with wt H7N9 (Figure 1E,F). The calculated LD_50_ of about 2 × 10^3^ PFU (Figure 4D) was 25-fold lower than the LD_50_ for the wt H7N9 virus (approximately 5 × 10^4^ PFU). The enhanced pathogenicity was also reflected by higher viral lung titers. Compared to H7N9-infected MxA^tg/tg^ mice, 100-fold higher lung titers of 5 × 10^6^ PFU were detected at three dpi with H7N9-PB2-E627K (Figure 4E). However, 10-fold lower lung titers of H7N9-PB2-E627K were observed in MxA^tg/tg^ mice after pretreatment with type I IFN one day prior to infection (Figure 4E). The already low lung titers in H7N9-infected MxA^tg/tg^ mice of around 5 × 10^4^ PFU further decreased to 1 × 10^4^ PFU after pretreatment with IFN.

Together, these results strongly suggest that acquisition of PB2-E627K enhanced viral replication of H7N9 both in the absence and the presence of antivirally active MxA. However, unlike for IAVs showing MxA resistance mutations in NP [12], IFN-pretreatment efficiently reduced lung titers of H7N9-PB2-E627K, suggesting that the enhanced MxA resistance of H7N9-PB2-E627K is due to an increased overall viral fitness rather than true MxA escape.

## 4. Discussion

With MxA being a major component of the species barrier, a hallmark of human-adapted seasonal and pandemic IAVs is the loss of MxA sensitivity. Here, we analyzed whether avian IAV H7N9 is able to escape from MxA restriction by allowing prolonged replication in MxA-transgenic Rag2^−/−^ mice. Although multiple mutations were acquired in almost all viral segments, most of these mutations were only present in low frequencies and therefore likely not responsible for the increased viral growth in the mice. Unexpectedly, none of the previously described MxA-resistance mutations were detected in the viral NP in our study. Instead, the virus seemed to exploit a different adaptation strategy by increasing viral polymerase activity. This was mainly accomplished by the fixation of the well-described E627K amino acid exchange in PB2, which is known to strongly enhance the viral replication of avian IAVs in mammalian cells [22] by enabling the use of mammalian co-factor ANP32A. It is therefore conceivable that the increased replication capacity enables these viruses to overrun the antiviral effect of MxA to some extent. Indeed, it has previously been reported that polymerase activity-enhancing mutations can even increase Mx resistance of an already human-adapted IAV [13].

Interestingly, PB2 627K was exclusively selected in MxA transgenic mice. However, in mice infected with d13′ virus isolates (Figure 3B,C), only about 50% of all sequencing reads encoded this amino acid. In these cases, other amino acid changes might have contributed to the increased replication efficiency in MxA transgenic mice. These could include amino acid substitution S534F in PB2, which is similarly abundant in these isolates and was previously reported to enhance polymerase activity in mammalian cells as well [24]. A second amino acid substitution of interest is NS1 S161T. Since the NS1 protein of IAV suppresses the expression of interferon-stimulated genes [25], the amino acid substitution S161T might increase this activity and thereby suppress MxA expression. S161T is located in the effector domain (ED) of NS1 and thus in close proximity to positions previously reported to be necessary for its IFN-antagonizing function [26].

MxA escape mutations found in human IAVs are largely absent in H7N9 isolates of both avian and human origin. In contrast, almost 60% of the isolates derived from H7N9 patients acquired PB2 E627K, while avian H7N9 strains lack this mutation with few exceptions [27]. This is in line with our observation that the E627K mutation was readily acquired in MxA-positive mice. To our surprise, when the initial isolates (Figure 2C) were checked for the presence of this mutation, PB2 E627K was only found in the isolates from infected MxA^tg/−^Rag2^−/−^ mice, whereas all remaining isolates of the MxA^−/−^ mice, with and without functional Rag2, lacked this amino acid substitution. This suggests that the E627K mutation might be preferentially acquired in the presence of antivirally active MxA. However, since it has also previously been shown that this mutation emerges when avian IAVs are passaged in MxA-negative BALB/c mice [28], it is currently unclear why this mutation was not acquired in the mice lacking MxA.

It has been previously shown that the acquisition of MxA escape mutations in the nucleoprotein is associated with a loss of fitness [11]. Thus, the acquisition of both the MxA escape mutations and the adaptive mutations required to compensate for the resulting attenuation might only be observed in very rare cases. This could explain the absence of MxA escape mutations in NP after infection of MxA^tg/−^Rag2^−/−^ mice with H7N9 and also in human-derived H7N9 isolates. The H7N9 isolate used in this study, A/Guangdong/17FS003/2016 (previously reported as A/Guangdong/Th008/2017), was obtained on the sixth day of infection [29,30]. Whether MxA escape mutations in NP could be acquired later during H7N9 infections is unclear. However, due to the absence of these mutations in the available H7N9 sequences, as well as in our results, it seems to be unlikely. The absence of the PB2 E627K mutation in 40% of human H7N9 isolates might be explained by early time points of virus isolation during infection or the presence of comparable mutations facilitating mammalian adaptation.

In summary, we showed that viral polymerase activity-enhancing mutations can partially overcome the antiviral activity of MxA against zoonotic H7N9. However, in patients and in our experimental infection model, no true MxA escape mutations were detected, suggesting that, fortunately, MxA still seems to pose a high human species barrier for H7N9. Due to the segmented genome of IAV, however, there is a constant risk of the acquisition of an already MxA-resistant NP gene from human-adapted viruses. We previously showed that H7N7 tolerates the incorporation of an MxA-resistant NP, thereby raising MxA resistance [10].

## Data Availability

Not applicable.
